# Alemtuzumab treatment for steroid-refractory acute graft-versus-host disease leads to severe immunosuppression but not to relapse of malignant disease

**DOI:** 10.1038/s41409-023-02144-8

**Published:** 2023-11-06

**Authors:** Lennart Philippen, Natalie Schub, Andreas Günther, Gunnar Cario, Martin Schrappe, Roland Repp, Martin Gramatzki

**Affiliations:** 1 Division of Stem Cell Transplantation and Immunotherapy, University Hospital Schleswig-Holstein and Christian-Albrechts-University Kiel, Kiel, Germany; 2 Department of Pediatrics, University Hospital Schleswig-Holstein and Christian-Albrechts-University Kiel, Kiel, Germany

**Keywords:** Haematological cancer, Bone marrow transplantation, Immunosurveillance, Immunotherapy, Allotransplantation

## To the Editor:

The fundamental therapeutic mechanism of allogeneic hematopoietic stem cell transplantation (HCT) in hematologic malignancies is the graft-versus-tumor reactivity mediated by donor T cells [1, 2]. While severe acute graft-versus-host disease (aGVHD) is a clinical situation to be avoided, patients affected tend to show fewer relapses [3]. Even so, aGVHD is the most relevant complication of allogeneic HCT and the mortality of steroid-refractory grade III and IV aGVHD is unacceptably high. The response to therapy after first-line treatment with glucocorticoids is still limited despite the advantage of recently introduced ruxolitinib [4, 5].

Previously, we and others have demonstrated that the pan-lymphoid CD52 antibody alemtuzumab is able to significantly improve steroid-refractory aGVHD [6–8]. However, this treatment usually causes complete lymphocyte depletion at least in the blood compartment [8, 9]. Severe immunosuppression may lead to infectious complications, particularly reactivation of cytomegalovirus. Furthermore, there are concerns that profound loss of immunosurveillance may facilitate an increased rate of relapse of the malignant disease that initially led to transplantation [10]. For prophylactic alemtuzumab application during conditioning, even a dose dependency regarding relapse and infection rate was suggested [11].

In this single-center analysis, the role of immunosurveillance early after HCT was evaluated in 54 consecutive patients transplanted for various hematologic malignancies who received alemtuzumab between June 2005 and October 2018 for steroid-refractory aGVHD of grade III or IV. Steroid refractoriness of aGVHD was defined as progress after 7 days of high-dose intravenous methyl-prednisolone equivalent. Patients had been diagnosed with acute myelogenous leukemia, myelodysplastic syndrome, myeloproliferative neoplasm, chronic myelomonocytic leukemia, chronic myelogenous leukemia, acute lymphoblastic leukemia, B- or T-cell non-Hodgkins lymphomas or multiple myeloma (Supplementary Table S1 provides patient details). The median age at the time of HCT was 55 years (range of 13–68 years). Sixteen patients (30%) received transplants from matched related donors (MRD), 22 patients (41%) of HLA-identical (10/10) matched unrelated donors (MUD) and 16 patients (30%) received non-HLA-identical (<10/10) stem cells from mismatched unrelated donors (MMUD). Twelve patients (22%) received a myeloablative conditioning treatment, and 39 patients (72%) received a reduced intensity conditioning regimen. Three patients (6%) were treated with a non-myeloablative conditioning regimen. While 7 (13%) patients (all with MRD) received no T-cell depletion as part of the conditioning regimen, in 42 (78%) patients anti-thymocyte globuline (ATG Fresenius, later Neovii Biotech, Gräfelfing, Germany) and in 4 (7%) patients alemtuzumab (Sanofi-Aventis, Frankfurt, previously Bayer (Schering), Leverkusen, Germany) were added. In one patient, post-transplantation cyclophosphamide was applied when transplanted from an HLA-B MMUD. Standard GVHD prophylaxis consisted of CsA and mycophenolat mofetil. A single patient received GVHD prophylaxis with methotrexate and CsA.

Treatment with alemtuzumab for steroid-refractory aGVHD asked for an absolute dose of 5–10 mg given on 1 or 2 days, followed by repeated doses alemtuzumab approximately every 14 days, usually for 6–8 weeks. As reported in detail [8], this dosing schedule has been established after initially patients received higher doses. It became apparent that due to limited lymphatic tissue expressing the CD52 antigen in these immunosuppressed patients in the early post-transplantation phase this dosing was sufficient. Once alemtuzumab was initiated, concurrent systemic immunosuppressive therapy was reduced step-wise, generally to CsA or tacrolimus only.

**Fig. 1:**
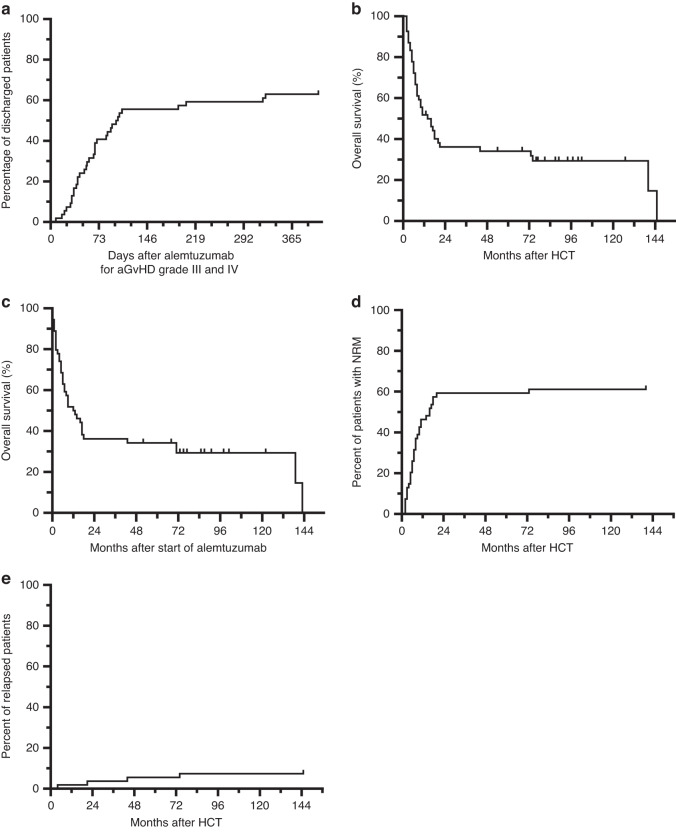
Clinical course and survival outcomes in patients (*n* = 54) treated by alemtuzumab for steroid-refractory grade III or IV aGVHD. **a** Percentage of patients that could be discharged from the hospital after start of alemtuzumab. **b** Kaplan–Meier survival curves are shown for overall survival from start of HCT or **c** from start of alemtuzumab. **d** Time-dependent description of patient events after HCT for non-relapse mortality (NRM) (*N* = 34) or **e** relapse (*N* = 5).

With extensive evaluation as required for gastrointestinal complications [12], in 50 of 54 patients, aGVHD was histologically confirmed by gastrointestinal biopsy and in 2 patients by liver histology. The median time between HCT and the first application of alemtuzumab for steroid-refractory aGVHD grade III and IV was 50 days (range of 20–180). The median total dose of alemtuzumab given was 27.5 mg (range of 2–191 mg). Thirty-five patients (65%) showed a meaningful response that led to clinical improvement, allowing discharge from the hospital after a median time of 65 days (range 8–405 days) after the first alemtuzumab (Fig. 1a). Only two patients (6%) needed a re-admission related to their GVHD soon after becoming an outpatient. Continuous immunosuppression, usually with a calcineurin inhibitor, was necessary for at least a month in a half of the cohort (18 patients, 50%) after discharge from the hospital. During follow-up, only 17 patients (49%) of the 35 outpatients that improved developed later symptoms of chronic GVHD (cGVHD) requiring medication.

Median follow-up time of surviving patients was 81 months. Fifteen patients (28%) were alive at the time of last follow-up. Median overall survival from the day of transplant for the whole patient cohort was 14 months (95% confidence interval (CI) 5.8–22.2) (Fig. 1b) or 12 months (95% CI 3.9–10.1) after start of alemtuzumab treatment (Fig. 1c). The probability of overall survival at 5 years after transplant was 34% (16 patients were still alive at this time point). Seventeen individuals died while still being in-patients due to their severe aGVHD due to non-relapse complications, mainly infections. Overall, 34 patients (65%) died because of non-relapse mortality (Fig. 1d). Only five patients relapsed (9%) (Fig. 1e). One patient with AML relapsed early and died 74 days after the first application of alemtuzumab given for steroid-refractory aGVHD. Four additional patients developed a late relapse, namely 17, 43, 71 and 143 months after the start of alemtuzumab.

One known side effect of clinical importance is the reactivation of CMV after alemtuzumab application. Thirty-five patients (65%) had CMV status positive for the donor and/or recipient. Three of these patients developed histologically confirmed CMV-mediated colitis and one patient a CMV-pneumonia.

To prevent severe aGVHD, many different strategies, such as graft selection and modifications, prophylactic immunosuppression as well as biologic markers for early detection and intervention, were developed. However, even with recently introduced drugs, particularly kinase-inhibitors such as ruxolitinib, in grade III and IV aGVHD the therapeutic effect is limited [4]. Alemtuzumab has long been used as part of the conditioning regimen to reduce alloreactivity. We and others have applied alemtuzumab treatment to diminish the cellular immune reactivity when needed in aGVHD [6–8] or cGVHD since it leads to almost complete depletion of lymphocytes in the blood and may alter the remaining lymphocyte subset composition thereafter [8, 9, 13]. Importantly, due to this intervention with alemtuzumab aGVHD could be reverted in approximately two thirds of the patients in the present study. Interestingly, in a significant number of patients, “re-booting” of the immune system apparently allowed them to regain appropriate immune functions without little or no signs of ongoing GVHD and required surprisingly limited ongoing immunosuppression.

The use of alemtuzumab in aGVHD rapidly diminishes immune cells and immune activity. This approach may lead to virus reactivation and clinically requires particular attention in situations where the patient and/or donor is CMV positive. It could be argued that with such an approach early after transplantation due to lack of immunosurveillance relapse may become a problem. However, we saw few relapses even in patients with unfavorable prognostic factors and without significant subsequent cGVHD. This would suggest that intense aGVHD can help to eradicate remaining tumor cells fast and completely. Thus, these findings provide evidence that tumor responses seen in severe aGVHD may be so profound that even the deep immunosuppression by alemtuzumab therapy would not be deleterious for tumor control. Then a second attempt of a more compatible immunoreconstitution may be a second chance for some of these severely ill patients.

### Supplementary information

Table 1

## Data Availability

Data of this work are available from the corresponding author upon reasonable request.
