# Supplementary material for: Alemtuzumab treatment for steroid-refractory acute graft-versus-host disease leads to severe immunosuppression but not to relapse of malignant disease
Source: Bone Marrow Transplant. 2023 Nov 6;59(1):153–5. doi: 10.1038/s41409-023-02144-8 (PMC10781631; doi:10.1038/s41409-023-02144-8)
Supplement: Supplementary file 1 — Table 1 [file 41409_2023_2144_MOESM1_ESM.docx]

**Suppl. Table 1. Patient characteristics and outcome**

| **Patient**  **number** | **Age** | **Disease** | **Conditioning regimen** | **Donor** | **GVHD grade** | **Alemtuzumab (in mg) *** | **CMV**  **(donor/ recipient)** | **Discharge from hospital after alemtuzumab (days) *** | **OS after HCT/ start alemtuzumab**  **(months)** | **Outcome at last-follow-up** |
| --- | --- | --- | --- | --- | --- | --- | --- | --- | --- | --- |
| 1 | 37 | CMML | Bu/Cy/ATG | MUD | IV | 153 | +/+ | - | 7 / 6 | NRM |
| 2 | 48 | AML | Bu/Cy | MRD | IV | 191 | +/+ | 93 | 9 / 7 | NRM |
| **3** | **47** | **MM** | **Flu/BCNU/Mel/ATG** | MUD | **III** | **91** | **+/-** | **24** | **145 / 143** | **relapse **** |
| 4 | 66 | AML | Flu/BCNU/Mel | MUD | IV | 106 | **+/+** | 321 | 140 / 139 | NRM |
| 5 | 42 | MM | Flu/BCNU/Mel | MRD | III | 16 | -/+ | 8 | 11 / 9 | NRM |
| **6** | **53** | **AML** | **Flu/BU/ATG** | **MRD** | **IV** | **100** | **-/-** | **-** | **74 / 71** | **relapse** |
| 7 | 63 | MDS | Flu/BU/ATG | MRD | III | 8 | +/+ | 32 | 127 / 122 | alive |
| 8 | 39 | AML | Bu/Cy | MRD | IV | 83 | -/- | 84 | 73 / 71 | NRM |
| 9 | 67 | MDS | Flu/BCNU/Mel/ATG | MUD | III | 83 | +/+ | 54 | 7 / 5 | NRM |
| 10 | 55 | CLL | Flu/Cy | MRD | IV | 63 | +/+ | - | 5 / 3 | NRM |
| **11** | **13** | **MDS** | **Bu/Cy/Mel/ATG** | **MMUD** | **III** | **33** | **+/+** | **91** | **44 / 43** | **relapse** |
| 12 | 54 | AML | Bu/Cy/ATG | MUD | IV | 53 | -/- | 108 | 8 / 7 | NRM |
| 13 | 52 | B-NHL | Flu/Cy | MRD | IV | 6 | -/+ | - | 2 / 1 | NRM |
| 14 | 53 | CML | Bu/Cy/ATG | MUD | IV | 82 | -/+ | 86 | 6 / 5 | NRM |
| **15** | **59** | **AML** | **Flu/TBI/ATG** | **MMUD** | **III** | **11** | **-/-** | **-** | **4 / 2** | **relapse** |
| 16 | 26 | AML | Bu/Cy/ATG | MUD | III | 6 | -/- | 17 | 89 / 87 | alive |
| 17 | 64 | MDS | Flu/BCNU/Mel/ATG | MRD | III | 17 | -/- | 32 | 97 / 91 | alive |
| 18 | 48 | ALL | TBI/Eto | MRD | III | 60 | **+/-** | 104 | 16 / 14 | NRM |
| 19 | 65 | B-NHL | Flu/Mel/ATG | MUD | IV | 10 | +/+ | - | 2 / 0 | NRM |
| 20 | 61 | CLL | Bu/Flu/Cy/ATG | MUD | IV | 33 | **+/-** | - | 2 / 1 | NRM |
| 21 | 22 | AML | Bu/Cy/ATG | MMUD | IV | 33 | -/- | 35 | 102 / 101 | alive |
| 22 | 42 | MDS | Flu/BCNU/Mel/ATG | MMUD | IV | 62 | -/- | - | 14 / 12 | NRM |
| 23 | 41 | AML | Bu/Cy/ATG | MUD | IV | 18 | -/- | 51 | 100 / 98 | alive |
| 24 | 60 | MDS | Flu/BCNU/Mel/ATG | MUD | III | 10 | -/+ | - | 5 / 0 | NRM |
| 25 | 64 | AML | Flu/BCNU/Mel/ATG | MRD | III | 18 | -/- | 44 | 81 / 77 | alive |
| 26 | 20 | AML | Bu/Cy/ATG | MMUD | III | 13 | -/+ | 67 | 87 / 85 | alive |
| 27 | 61 | B-NHL | Bu/Flu/Cy/ATG | MUD | III | 8 | -/+ | 21 | 94 / 91 | alive |
| 28 | 55 | AML | Flu/Mel/ATG | MRD | III | 18 | -/- | 67 | 76 / 73 | alive |
| 29 | 57 | AML | Flu/BCNU/Mel/ATG | MRD | III | 25 | **+/-** | 405 | 8 / 6 | NRM |
| **30** | **44** | **AML** | **Flu/BCNU/Mel/Alt** | MMUD | **III** | **45** | **-/-** | **193** | **21 / 17** | **relapse** |
| 31 | 62 | MDS | Flu/BCNU/Mel/ATG | MMUD | III | 15 | -/+ | 39 | 7 / 6 | NRM |
| 32 | 49 | T-NHL | BEAM/Alt | MUD | IV | 2 | -/+ | - | 6 / 2 | NRM |
| 33 | 44 | CLL | Bu/Flu/Cy/ATG | MMUD | IV | 45 | -/+ | - | 2 / 1 | NRM |
| 34 | 65 | AML | Flu/BCNU/Mel/ATG | MMUD | III | 20 | +/+ | 35 | 73 / 68 | alive |
| 35 | 68 | AML | Flu/BCNU/Mel/ATG | MUD | IV | 8 | -/+ | 99 | 68 / 68 | alive |
| 36 | 56 | CLL | Bu/Flu/Cy/ATG | MMUD | IV | 43 | -/+ | - | 3 / 2 | NRM |
| 37 | 42 | AML | Flu/BCNU/Mel/ATG | MRD | IV | 25 | +/+ | 102 | 77 / 75 | alive |
| 38 | 50 | T-NHL | BEAM/Alt | MMUD | III | 33 | -/+ | - | 8 / 2 | NRM |
| 39 | 43 | AML | Flu/BCNU/Mel/ATG | MUD | III | 60 | +/+ | 70 | 18 / 17 | NRM |
| 40 | 40 | AML | Flu/BCNU/Mel/ATG | MMUD | III | 45 | -/- | - | 3 / 2 | NRM |
| 41 | 63 | AML | Flu/BCNU/Mel | MRD | IV | 25 | -/- | 67 | 18 / 17 | NRM |
| 42 | 63 | MPN | Flu/BCNU/Mel/ATG | MUD | IV | 19 | -/+ | - | 4 / 4 | NRM |
| 43 | 18 | AML | Flu/BCNU/Mel/ATG | MMUD | IV | 10 | -/- | 41 | 54 / 52 | alive |
| 44 | 64 | MDS | Flu/BCNU/Mel/ATG | MRD | III | 18 | -/- | 30 | 10 / 9 | NRM |
| 45 | 66 | MDS | Flu/BCNU/Mel/ATG | MUD | IV | 5 | +/+ | - | 3 / 0 | NRM |
| 46 | 66 | AML | Flu/BCNU/Mel/ATG | MMUD | IV | 48 | +/+ | - | 17 / 16 | NRM |
| 47 | 67 | MDS | Flu/BCNU/Mel/ATG | MUD | IV | 65 | -/+ | - | 10 / 8 | NRM |
| 48 | 60 | MM | Flu/Treo/ATG | MRD | IV | 30 | -/+ | 58 | 5 / 4 | NRM |
| 49 | 61 | MPN | Flu/Treo/ATG | MUD | IV | 40 | +/+ | 205 | 16 / 13 | NRM |
| 50 | 50 | AML | Flu/BCNU/Mel/ATG | MUD | IV | 23 | +/+ | 41 | 20 / 18 | NRM |
| 51 | 19 | AML | Flu/BCNU/Mel/ATG | MMUD | IV | 20 | +/+ | 65 | 17 / 16 | alive |
| 52 | 53 | T-PLL | BEAM/Alt | MUD | III | 60 | -/- | 325 | 13 / 12 | alive |
| 53 | 59 | CLL | Flu/Cy/ATG | MUD | IV | 45 | -/- | - | 11 / 9 | NRM |
| 54 | 62 | B-NHL | Thio/Flu/Bu/Post-Tx Cy | MMUD | IV | 15 | -/- | 55 | 6 / 5 | NRM |

* applied for aGVHD, ** patient who relapsed are highlighted in bold.

ALL acute lymphoblastic leukemia, Alt alemtuzumab used as part of the conditioning, AML acute myelogenous Leukemia, ATG anti-thymocyte-globulin, B-/T-NHL B-/T-cell non-Hodgkin lymphoma, BCNU carmustine, BEAM carmustine, etoposide, cytosine arabinoside, melphalan, Bu busulfan, CLL chronic lymphocytic leukemia, CML chronic myelogenous leukemia, CMML chronic myelomonocytic leukemia, Cy cyclophosphamide, Flu fludararbine, MDS myelodysplastic syndrome, Mel melphalan, MM multiple myeloma, MPN myeloproliferative neoplasm, MMUD mismatched unrelated donor, MRD matched related donor, MUD matched unrelated donor, NRM non-relapse mortality, OS overall survival, post-Tx Cy post-transplantation cyclophosphamide, TBI total body irradiation, T-PLL T-prolymphocytic leukemia, Thio thiotepa, Treo treosulfan
